# Secretory IgA *N*-glycans contribute to the protection against *E. coli* O55 infection of germ-free piglets

**DOI:** 10.1038/s41385-020-00345-8

**Published:** 2020-09-24

**Authors:** Leona Raskova Kafkova, Diana Brokesova, Michal Krupka, Zuzana Stehlikova, Jiri Dvorak, Stepan Coufal, Alena Fajstova, Dagmar Srutkova, Katerina Stepanova, Petra Hermanova, Renata Stepankova, Ivo Uberall, Jozef Skarda, Zdenek Novak, Luca Vannucci, Helena Tlaskalova-Hogenova, Zuzana Jiraskova Zakostelska, Marek Sinkora, Jiri Mestecky, Milan Raska

**Affiliations:** 1 Department of Immunology, Faculty of Medicine and Dentistry, Palacky University Olomouc, Olomouc, Czech Republic; 2 Laboratory of Cellular and Molecular Immunology, Institute of Microbiology of the Czech Academy of Sciences, Prague, Czech Republic; 3 Laboratory of Gnotobiology, Institute of Microbiology of the Czech Academy of Sciences, Novy Hradek, Czech Republic; 4 Department of Clinical and Molecular Pathology, Faculty of Medicine and Dentistry, Palacky University Olomouc, Olomouc, Czech Republic; 5 Department of Surgery, University of Alabama at Birmingham, Birmingham, AL USA; 6 Laboratory of Immunotherapy, Institute of Microbiology of the Czech Academy of Sciences, Prague, Czech Republic; 7 Department of Microbiology, University of Alabama at Birmingham, Birmingham, AL USA

## Abstract

Mucosal surfaces are colonized by highly diverse commensal microbiota. Coating with secretory IgA (SIgA) promotes the survival of commensal bacteria while it inhibits the invasion by pathogens. Bacterial coating could be mediated by antigen-specific SIgA recognition, polyreactivity, and/or by the SIgA-associated glycans. In contrast to many in vitro studies, only a few reported the effect of SIgA glycans in vivo. Here, we used a germ-free antibody-free newborn piglets model to compare the protective effect of SIgA, SIgA with enzymatically removed *N*-glycans, Fab, and Fc containing the secretory component (Fc-SC) during oral necrotoxigenic *E. coli* O55 challenge. SIgA, Fab, and Fc-SC were protective, whereas removal of *N*-glycans from SIgA reduced SIgA-mediated protection as demonstrated by piglets’ intestinal histology, clinical status, and survival. In vitro analyses indicated that deglycosylation of SIgA did not reduce agglutination of *E. coli* O55. These findings highlight the role of SIgA-associated *N*-glycans in protection. Further structural studies of SIgA-associated glycans would lead to the identification of those involved in the species-specific inhibition of attachment to corresponding epithelial cells.

## Introduction

Large surfaces of mucosae, especially of the intestinal tract, are colonized by enormous numbers of highly diverse microbiota.^1^ A complex interplay of complementary mechanisms of innate and specific immunity contains this microbiota in its physiological compartment without compromising the integrity of mucosal tissues.^2^ In vivo bacteria are coated with mucosal antibodies, in humans dominantly of the IgA isotype,^2–6^ that provide protection against pathogenic microorganisms but at the same time promote successful survival and containment of endogenous commensal mucosal microbiota.^3–6^ However, it is not known whether this coating of bacteria is exclusively dependent on the highly specific antibody activity, polyreactivity, and the interactions of IgA-associated glycans with complementary structures on microorganisms.^5,7–16^ Considering the enormous numbers and highly variable species diversity along with the large number of potential antigenic determinants on bacterial structures, it is probable that all three mechanisms participate in the protective activity of secretory IgA (SIgA).^2–5,8,17,18^ We reported that the binding of gram-negative bacteria to the glycan receptors on epithelial cells is inhibited by IgA-associated glycans as confirmed and extended to other species of both gram-positive and gram-negative bacteria.^10,17,19–22^ In addition to the heavy (H) α chains of IgA, secretory component (SC), the extracellular part of the polymeric Ig receptor (pIgR), is heavily glycosylated and acts as a highly effective inhibitor of bacteria adherence to epithelial cells^7,10,13,14,19,20,23,24^ thus enforcing the protective functions of SIgA.^8,25^ Structural studies of human SIgA as well as monoclonal myeloma IgA proteins revealed a remarkable degree of glycan heterogeneity with respect to the composition, number, site of attachment and primary structures of the glycan side-chains.^9,19,20,26–28^ Whether these glycan-dependent interactions are of importance in the protection in vivo has been previously demonstrated only in the murine model of protection of the respiratory tract against *Shigella flexneri* in which recombinant glycosylated SC bound to Shigella-specific IgA was tested.^29^

In our studies reported herein we exploited a unique model of germ-free, colostrum- or milk-deprived newborn piglets which, in sharp contrast to humans, mice, rats, or rabbits, are born without any transplacentally acquired antibodies due to the epitheliochorial structure of the placenta.^30,31^ Protective antibodies are acquired postnatally from colostrum and milk rich in their Ig content. Thus, deprivation of newborn piglets of colostrum and milk results in the fatal infection^32–35^ not only in laboratory but also under standard farm conditions. In preliminary germ-free piglets model, we observed that SIgA antibodies derived from human milk or colostrum display their protective effect against challenges with *E. coli*. However, the mechanisms involved in the Ig-mediated protection were not addressed. The availability of large quantities of human SIgA and the precise characterization of its structure, particularly of its associated glycans^9,19,20,26,28^ and a highly relevant piglet experimental model, permitted us to perform studies addressing the role of specific antibody activity and IgA glycan-dependent activity against necrotoxigenic *E. coli* 055:H4 (further abbreviated as *E*. *coli* O55), which in germ-free piglets cause fatal infection.^36^ We prepared SIgA, SIgA with enzymatically removed *N*-glycans, and two fragments of SIgA obtained by the cleavage with hinge region-specific protease, composed of IgA Fc fragment with SC and J chain designated as Fc-SC, and fragment corresponding to SIgA Fab, and we compared their protective activity against *E. coli* O55 oral challenge of germ-free newborn piglets.

The generated results demonstrated that the IgA-associated glycans play an important protective role in vivo as demonstrated by a substantial reduction of SIgA protection after *N*-glycans removal.

## Results

### Preparation and characterization of SIgA, deglycosylated SIgA and SIgA fragments

**Fig. 1:**
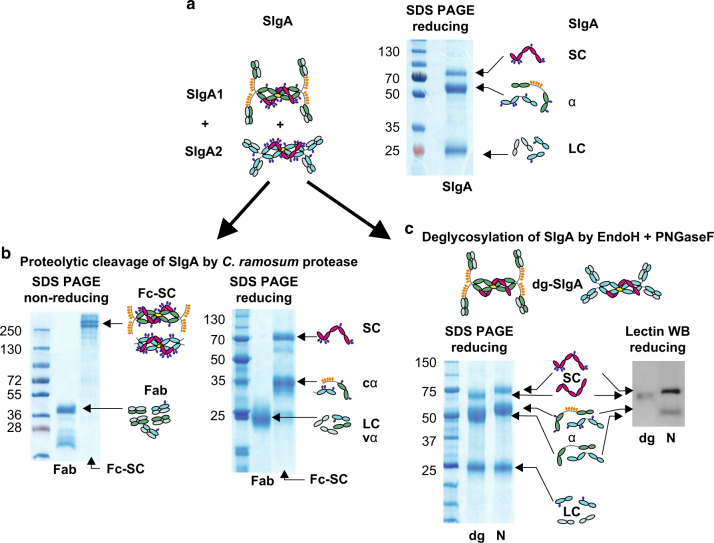
IgA preparations tested throughout piglet’s experiments. Pooled human colostrum/milk served as the source of SIgA. **a** Gel-permeation chromatography-purified SIgA was separated by SDS PAGE stained by Coomassie Blue. **b** SIgA digested with *C. ramosum* protease generates a large SIgA fragment designated Fc-SC (containing Fc portions of IgA plus SC plus J chain) and Fab fragment. Fc-SC and Fab were separated by gel-permeation chromatography and analyzed by SDS PAGE under non-reducing and reducing conditions to identify components of Fc-SC (SC and constant region—cα) and Fab (LC light chain and vα variable region). **c** SIgA deglycosylated by EndoH and PNGaseF was designated dg-SIgA and characterized by the mobility shift on SDS PAGE in comparison to untreated (N) SIgA (left part of **c**) and by reduction of reactivity with high mannose-specific lectin from *Galanthus nivalis* on Western blot (right part of **c**).

SIgA was purified by gel-permeation chromatography from a protein-enriched fraction of pooled human colostrum/milk prepared by ammonium sulfate precipitation.^37^ To confirm purity and integrity of the preparation, SIgA was characterized by SDS PAGE, and molecular components were identified (Fig. 1a). Subsequently SIgA was digested by recombinant IgA protease from *Clostridium ramosum* that cleaves both SIgA1 and SIgA2 at the *N*-terminus of the hinge region.^38^ The SIgA fragment containing Fc portions, J chain, and the secretory component was designated Fc-SC. The remaining fragment was Fab. The composition of generated fragments was analyzed by non-reducing SDS PAGE where Fc-SC exhibited the molecular mass of ≈250 kDa and Fab ≈40 kDa (Fig. 1b), and by reducing SDS PAGE where Fc-SC segregated into SC (≈75 kDa), *C. ramosum*-cleaved α chain constant domains of IgA1 and IgA2 (cα ≈ 35 and 40 kDa, respectively), and J chain (≈16 kDa) (Fig. 1b, right subpanel). To prepare deglycosylated form of SIgA (designated dg-SIgA), *N*-linked glycans on SIgA were removed by the sequential cleavage by EndoH followed by PNGaseF. This leads to ≈2.5–6 kDa reduction in the mol. mass of the α chain and ≈14–20 kDa reduction of SC, according to previously reported analyses.^10,20,39^ The efficiency of *N*-glycans removal was determined by the shift of mobility of HC and SC on SDS PAGE (Fig. 1c, left subpanel) and by the reduction in reactivity with high mannose *N*-glycans-specific lectin from *Galanthus nivalis* on Western blot (Fig. 1c, right subpanel).

### Infection of piglets with E. coli O55 preincubated with various molecular SIgA forms

**Fig. 2:**
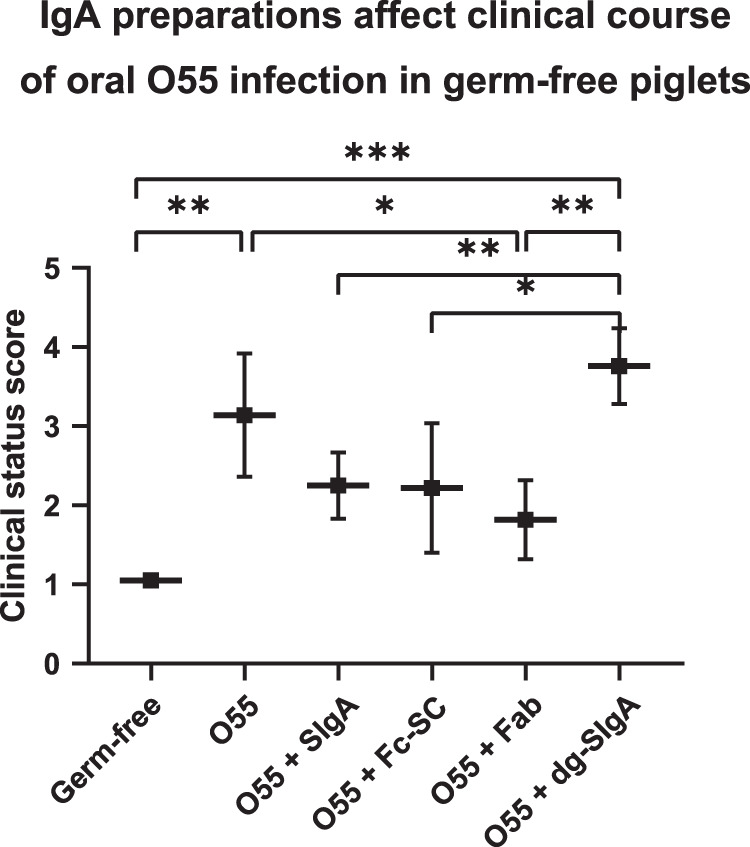
Evaluation of clinical status of piglets challenged with O55 *E. coli* incubated with various SIgA forms. Germ-free piglets (five piglets per group) at day 5 after delivery were challenged orally with *E. coli* O55 preincubated with SIgA forms or PBS. Individual groups of five piglets were labeled based on *E. coli* O55 pretreatment. Germ-free–*E. coli*-unexposed piglets. 35 h after challenge clinical status was evaluated as weighted means of scaled parameters: temperature, drink volume, stool, and interest as specified in Table 2 and Supplementary Table 1. Means ± SD are provided. *P* values were calculated using Kruskal-Wallis test with Dunn-Bonferroni post-hoc test. *p < 0.05, **p < 0.01, ***p < 0.001.

Piglets were orally infected with 4 × 10^5^ CFU of *E. coli* O55 preincubated with individual IgA preparations (Fig. 1) at doses equimolar to 20 mg of SIgA (Table 1). The clinical status was evaluated 35 h later as weighted mean of temperature, volume per feeding, stool quality, and viability scaled according to Table 2. It is summarized in Fig. 2. Evaluation of individual piglets is described in Supplementary Table 1. The most favorable clinical status was evident in the group of piglets infected by *E. coli* O55 preincubated either with SIgA or Fc-SC, or Fab. In contrast, the worst clinical status was observed in group of piglets infected with *E. coli* O55 preincubated with dg-SIgA.

**Table 1:** Preparation of SIgA forms treated *E. coli* O55 inoculum.

Group	Dose per individual piglet					
		SIgA preparation				
	O55 [CFU]	SIgA [mg]	Fab [mg]	Fc-SC [mg]	dg-SIgA [mg]	Note
Germ-free	–	–	–	–	–	
O55	4 × 10^5^	–	–	–	–	
O55 + SIgA	4 × 10^5^	20	–	–	–	
O55 + Fc-SC	4 × 10^5^	–	–	12	–	
O55 + Fab	4 × 10^5^	–	8	–	–	
O55 + dg-SIgA	4 × 10^5^	–	–	–	18	

Samples were prepared for each group individually (five piglets per group) by dilution of individual components *E. coli O55* and SIgA preparation in sterile 5% glucose. These preparations were divided and used for piglet oral inoculation, followed by feeding with milk *ad libitum*.

**Table 2:** Clinical status evaluation in experimental piglets.

Scale	Scaled parameter			
	Temperature	Volume per feeding	Stool	Interest
1	normal range 36.7–37.7 °C	≥15 ml	Normal	Active movement, active response to stimuli
2	enhanced temperature 37.8–39.5 °C	<15 ml	Diarrhea	Still standing, weak response to stimuli
3	fever 39.5–41 °C	0 ml	–	Lying, lethargy
4	temperature declining < 39.5 °C	–	–	Trembling
5	hypothermia < 36.7 °C	–	–	Death

Each parameter (temperature, volume per feeding, stool quality, interest) was scored independently for each piglet (Supplementary Table 1) and overall clinical status was evaluated as weighted mean for each piglet. The weight for temperature was 0.4, for drink volume 0.2, for stool 0.2, and for interest 0.4.

### Histological evaluation of intestinal tissues

**Fig. 3:**
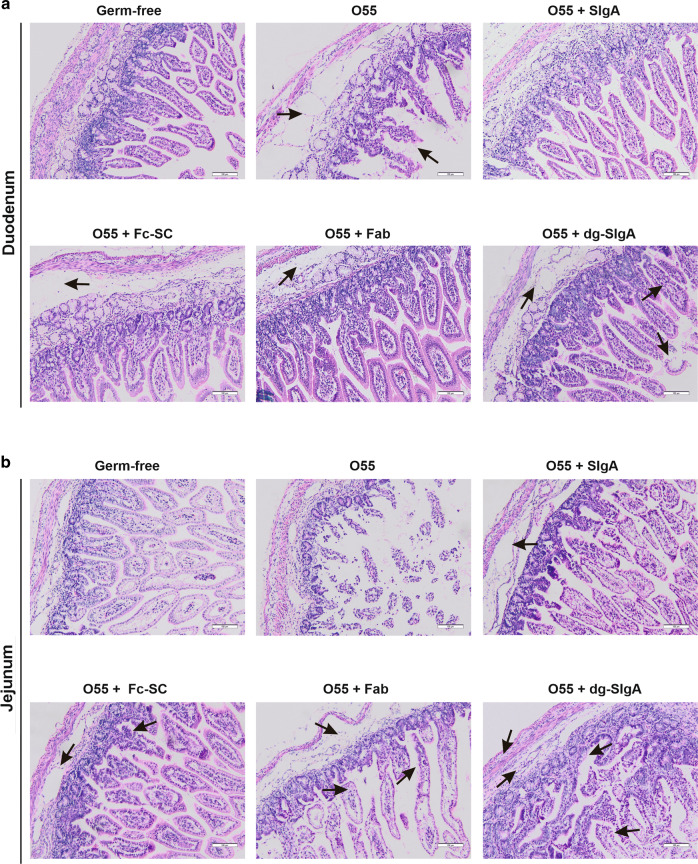

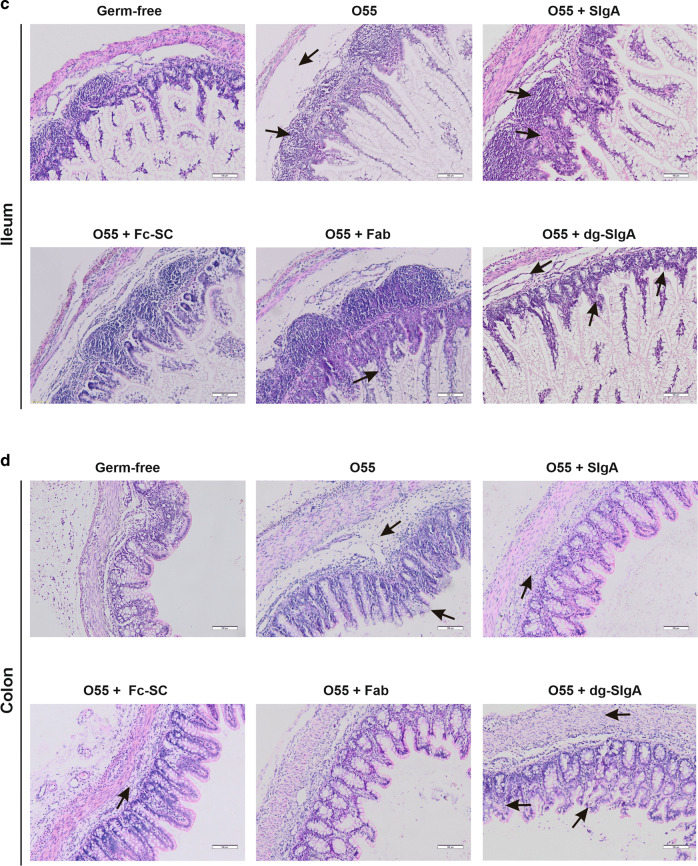
Histological features of intestine samples from experimental piglets. Groups are specified according to the treatment as in the Fig. 2. Characteristic alteration from normal histology are specified. *E. coli* O55 is abbreviated as O55. **a** Duodenum. O55—edematous lamina propria, villi with apical destruction of epithelium. Reduced count of vacuolated enterocytes. O55 + SIgA—normal histology with minimal edema in lamina propria. O55 + Fc-SC—focal moderate edematous lamina propria. O55 + Fab—moderate edema of the lamina propria. O55 + dg-SIgA—edematous lamina propria, villi congested and apical destruction of epithelium. Reduced count of vacuolated enterocytes. **b** Jejunum. O55—lamina propria with focal inflammatory cellularization, destructed villi and crypt epithelia and stroma. O55 + SIgA—moderate edema in lamina propria and villi congestion. O55 + Fc-SC—moderate edema of lamina propria, congestion of crypts. O55 + Fab—edematous lamina propria, moderate congestion and leukocytosis in villi stroma, minute focal changes in epithelial lining. O55 + dg-SIgA—edematous lamina propria, stroma of villi preserved, but prominent destruction of epithelial lining of villi and crypts. **c** Ileum. O55—edematous lamina propria, moderate activation of the lymphoid tissue. O55 + SIgA—moderate crypt hyperplasia, activation of lymphoid tissue. O55 + Fc-SC—normal histology. O55 + Fab—crypt and villi congestion and lymphocytosis. O55 + dg-SIgA—edematous lamina propria, crypt hyperplasia and epithelia erosions. **d** Colon. O55—heavily edematous lamina propria, enterocytes destruction in apical portion of villi. O55 + SIgA—moderate edema and enhanced cellularity of lamina propria, otherwise normal histology. O55 + Fc-SC—minute edema and cellularity of lamina propria, otherwise normal histology. O55 + Fab—normal histology. O55 + dg-SIgA—edematous and hypercellular lamina propria, congestion of villous stroma, focal apical enterocytes desquamation.

To determine the mechanisms involved in the development of clinical sign of infection, we performed histological analyses of duodenum, jejunum, distal ileum, and colon. We evaluated edema and cellular infiltration of lamina propria, crypts and villi morphology, and the presence of vacuolated epithelia cells on H&E-stained tissue sections (Fig. 3). Untreated germ-free piglets exhibited normal histology. Most prominent changes were identified in groups of piglets infected with *E. coli* O55 alone or *E. coli* O55 preincubated with dg-SIgA. In both groups deterioration of crypt epithelial cells and lamina propria edema was apparent in the duodenum, jejunum, ileum, and colon. Piglets infected with *E. coli* O55 preincubated with SIgA or with Fab exhibited no or minimal alterations of the duodenum, jejunum, ileum, and colon epithelia, crypts, and villi. Nevertheless, moderate lamina propria edema was detected in individual sections from all animals. In some sections from the piglets infected with *E. coli* O55 preincubated with Fab, moderate congestion and leukocytosis in villi stroma and minute changes in epithelial lining of jejunum were detected.

Reactive lymphoid hyperplasia determined as enlargement of Peyer’s patches detected by measurement of their size, was apparent in the ileum of piglets infected with *E. coli* O55 preincubated with SIgA, Fc-SC, and Fab. The epithelia destruction in ileum was generally less prominent than in other sections. In the groups of piglets infected by *E. coli* O55 alone and preincubated with dg-SIgA, colonic crypt hyperplasia and especially erosions of apical epithelial cells were the most dominant observations.

Vacuolated enterocytes were present in the duodenum sections from all animals, but clear reduction was observed in the group of piglets infected with *E. coli* O55 or *E. coli* O55 preincubated with dg-SIgA. Because vacuolated enterocytes are engaged in the absorption of Ig as well as other colostral or foreign proteins and glycoproteins from the gut lumen into the circulation,^40–43^ we examined the presence of human IgA in epithelial cells in frozen sections of duodenum from piglets exposed to *E. coli* O55 preincubated with SIgA (Fig. S1). IgA was identified on the tips of intestinal villi whereas intestinal crypts were negative. Furthermore, we examined sera of these animals for the presence of human IgA. Only trace amounts (<0.1 µg/ml) were detected after oral administration of 20 mg SlgA (Fig. S2).

### Bacterial load analyses in the intestine and other internal organs

**Fig. 4:**
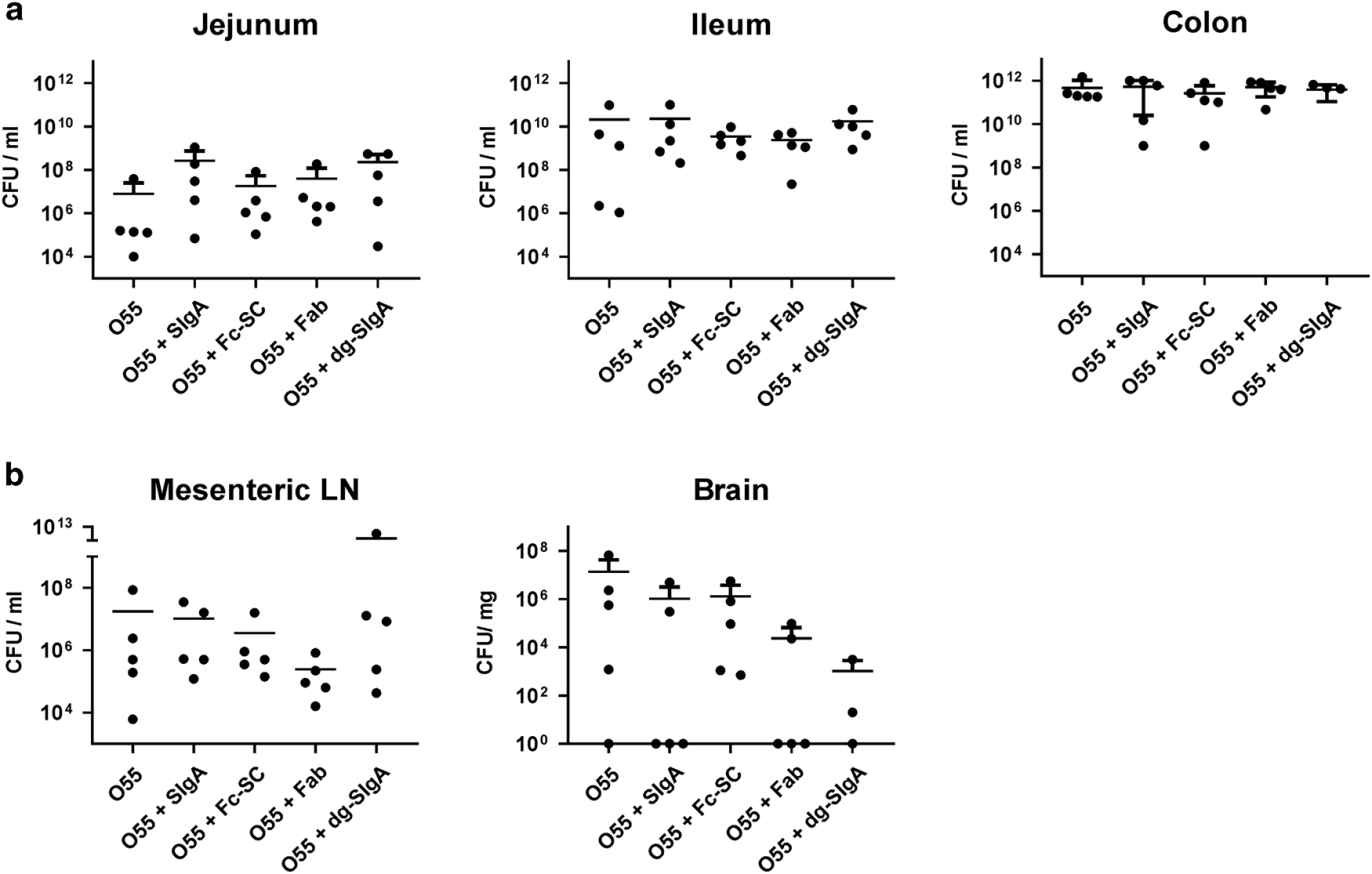
Bacterial load in various anatomical locations of *E. coli* O55 infected piglets. Bacterial load was determined after death from tissue samples as bacterial titer (CFU/ml of intestine content or CFU/mg of tissue). **a** Bacteria cultivated form jejunum, ileum and colon. **b** Bacteria cultivated from mesenteric lymph nodes (LN) and brain. In the case of brains from piglets infected with *E. coli* O55 preincubated with dg-SIgA only three piglet’s brains were tested for *E. coli*. None differences among groups were calculated significant.

At the time of the piglets’ euthanasia, the bacterial load in jejunum, ileum, colon, mesenteric lymph nodes, and brain was determined (Fig. 4). In the intestinal sections, no significant differences were detected among individual groups of piglets in jejunum, ileum, and colon samples. Instead, uniform CFU mean values rising along the jejunum to colon axis starting from 10^7^–10^8^ CFU/ml in the jejunum to 10^12^ CFU/ml in the colon was observed. In the mesenteric lymph nodes the lowest mean CFU was detected in *E. coli* O55 preincubated with Fab and highest CFU in dg-SIgA group; nevertheless, the enormous bacterial load variability (10^4^ CFU/mg–10^13^ CFU/mg) prevents the identification of any statistically significant differences. Furthermore, we performed analyses of stool concerning the bacterial counts and IgA1 and IgA2 concentrations. In general, analysis of *E. coli* O55 in the stool exhibited high variability of CFU which could be affected by nutrients availability according to the actual volume of ingested milk formulation before and during the experiment, by intestine passage speed, by volume of stool, and by individual variability of immune defense in outbred animals. All these components differ not only within individual groups, but also among individual groups of piglets since the clinical status of experimental groups differs. Nevertheless, there is a clear trend indicating the increase in the number of bacteria during the experiment course in all animals independently of treatment (Fig. S3A). This agrees with expected continuous reduction of SIgA or its derivatives in the intestine content during the course of experiment. Analysis of IgA1 and IgA2 subclasses in the stool of O55 + SIgA- and O55 + dg-SIgA-infected piglets indicates also high interindividual variability but the observed trend and the estimated area under curve indicates that SIgA1 is less stable in our experimental setup in comparison to SIgA2 (Fig. S3B). Further comparison of IgA1 and IgA2 in group of piglets infected by *E. coli* O55 incubated with SIgA or with dg-SIgA during sampling intervals 9 and 24 h after infection did not indicate substantially reduced stability after SIgA deglycosylation.

### Cytokine expression by the jejunum and ileum tissue

**Fig. 5:**
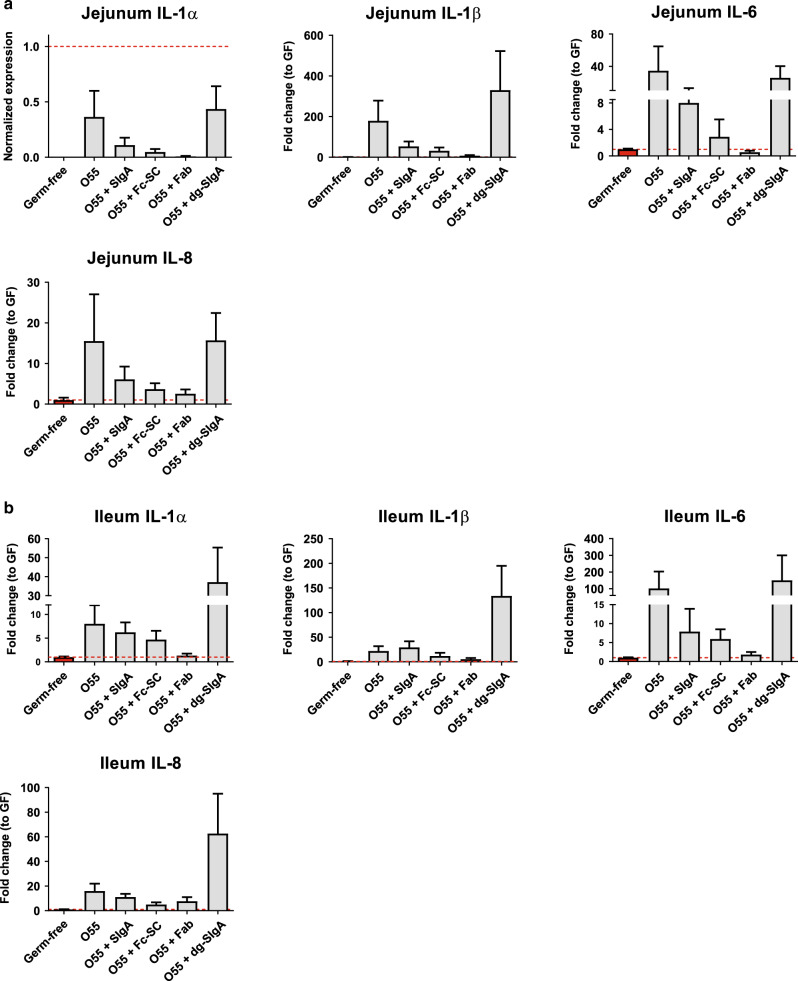
Cytokine expression in jejunum and distal ileum of infected piglets. The level of expression of pro-inflammatory cytokines was determined by quantitative PCR in (**a**) Jejunum and (**b**) Ileum. O55 increases gene expression of all determined pro-inflammatory cytokines in both jejunum and ileum. Exception is TNF-α and TGF-β in ileum, where no increase was noted. Further the treatment of *E. coli* O55 with either SIgA, Fc-SC, or Fab results in lower increase of pro-inflammatory cytokines gene expression in comparison with O55 although statistical singinficance was borderline. Similarly, treatment by *E. coli* pre-incubated with dg-SIgA results in increase of pro-inflammatory cytokines gene expression in comparison with O55 with borderline significance. Gene expression statistics were calculated using One-way ANOVA showing borderline significance between piglet groups for ileum expression of IL-1α *p* = 0.084, IL-1β *p* = 0.03, and IL-8 *p* = 0.083. Pair analysis using post-hoc Tukey test identified significant differences of ileum IL-1β expression between piglets challenged with *E. coli* O55 incubated with dg-SIgA versus Fab (*p* < 0.05) and borderline difference for dg-SIgA versus Fc-SC incubated groups (*p* = 0.051).

Because the gut histology demonstrated signs of inflammation and cellular damage, we determined mRNA levels of pro-inflammatory cytokines IL-1α, IL-1β, IL-6, and IL-8 in jejunum (Fig. 5a) and ileum (Fig. 5b) by RT-qPCR analysis. Although not statistically significant by the averages (due to high inter-animal variation among individual outbred piglets and the limited number of piglets—five per group), piglets in individual groups expressed a similar pattern that agrees with the clinical status (compare with Fig. 2). Higher levels of all inflammatory cytokines were recorded in piglets infected with *E. coli* O55 alone or with *E. coli* O55 preincubated with dg-SIgA, while lower values of inflammatory cytokines expression were detected in piglets infected with *E. coli* O55 preincubated either with SIgA, Fc-SC, or Fab. Therefore, intestine inflammation monitored by local inflammatory cytokines expression corresponds to histological analyses and is in agreement with the overall clinical course of infection.

### Interaction of SIgA preparations with E. coli O55

**Fig. 6:**
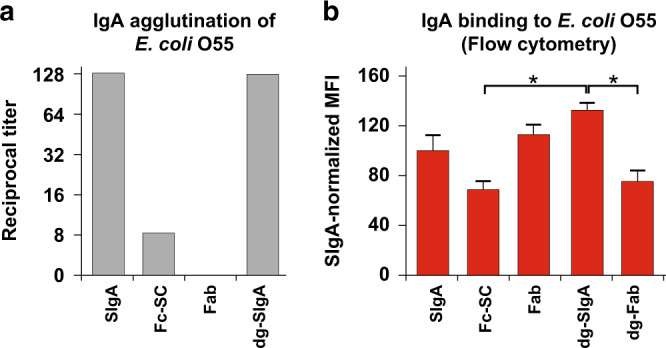
Interaction of IgA preparations with *E. coli* O55. **a** Individual fractions and modifications of SIgA at 2 mg/ml were titrated and incubated with 2 × 10^9^ CFU/ml of *E. coli* O55 at 37 °C followed by overnight incubation at 4 °C. The reciprocal of the highest dilution giving visible agglutination is presented as agglutination titer. Resulting values are from three independent experiments. **b** *E. coli* O55 were incubated with SIgA preparations conjugated with DyLight 488, washed, and analyzed by flow cytometry. After Fab deglycosylation, binding to *E. coli* O55 was reduced to 67% of its original value. Means ± SD are provided. *P* values were calculated using Kruskal-Wallis test with Dunn-Bonferroni post-hoc test. *p < 0.05.

To explain observed differences in the clinical course of the infection induced by *E. coli* O55 preincubated with individual SIgA preparations, we analyzed the interaction of all tested SIgA forms with *E. coli* O55 in vitro (Fig. 6). In micro-agglutination assay (Fig. 6a) native SIgA agglutinated *E. coli* O55 at highest dilution, Fc-SC exhibited reduced agglutination titer, and Fab fragment alone, due to its monovalency, did not agglutinate. Fab interaction with *E. coli* O55 was further supported by the observation, that removal of *N*-glycans from SIgA did not change the agglutination as compared to intact SIgA (Fig. 6a). The involvement of mannose-rich *N*-glycans variant on SIgA was specifically tested by the agglutination assay in the presence of 2.5% α-D-mannose,^20^ which in the case of our SIgA preparation and *E. coli* O55 carrying FimH39 variant of mannose-specific type 1 pili, did not affect the agglutination titer (Fig. S4).

To characterize the interaction of SIgA and its variants with *E. coli* O55 independently of binding valency, we used flow cytometry analysis in which individual SIgA preparations were conjugated with DyLight 488 fluorophore (Fig. 6b and S5). Fab bounds to *E. coli* O55 with the intensity similar to SIgA. In contrast, Fab-devoid Fc-SC fragment recognized *E. coli* O55 about 30% less intensively than SIgA, although the significance of this observation was not confirmed (Fig. 6b), indicating that both Fab-dependent and Fab-independent mechanisms are involved. Interestingly, dg-SIgA exhibited the strongest binding, significantly higher than Fc-SC.

Fab fragment of IgA2 subclass could contain one to two *N*-linked glycans^44^ that may contribute to Fab binding to *E. coli* pili. Thus, we further compared the binding of intact Fab and deglycosylated Fab (dg-Fab) with *E. coli* O55. Deglycosylation reduced insignificantly Fab binding (Fig. 6b), which could be due to the characteristic content of milk-unique combination of IgA1 and IgA2 subclasses (the ratio of SIgA1:SIgA2 in our preparation was 3:2), of which IgA1 Fab contains no *N*-linked glycans, IgA2m(1) contains one and IgA2m(2) and IgA2(n) contain two *N*-glycans per Fab^44^ and various proportion of high mannose and complex *N*-glycans on individual IgA Fc.^20^ In conclusion, our results indicate that (1) SIgA to *E. coli* O55 binding determined by flow cytometry was weak but variation was observed dependent on the presence of Fab-arms but not glycans and (2) O55 agglutination was dependent on the multimeric nature of SIgA and was independent of glycans.

### Survival of piglets infected by E. coli O55 preincubated with SIgA and dg-SIgA

**Fig. 7:**
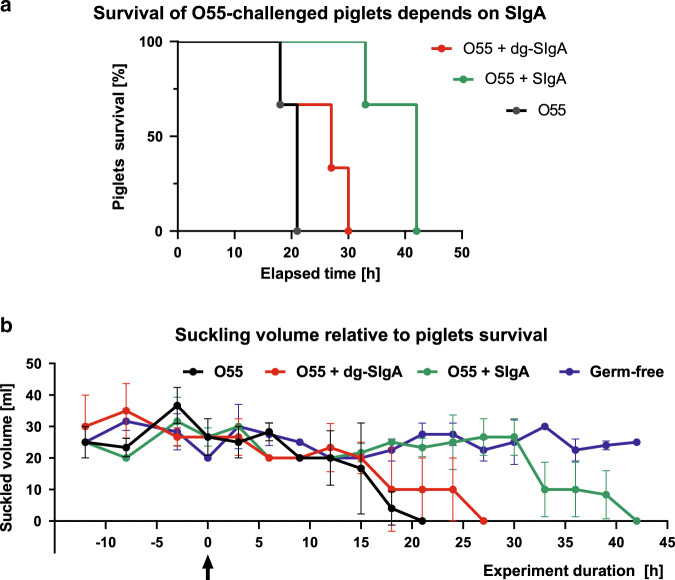
Survival of piglets treated by SIgA and deglycosylated SIgA. Germ-free piglets (three animals per group) at day 5 after delivery were either left unaffected or challenged orally with untreated *E. coli* O55 or *E. coli* O55 preincubated with SIgA, or dg-SIgA using the same dosage as in first experiment. Individual groups were labeled: Germ-free–control germ-free piglets; O55—piglets challenged orally with *E. coli* O55; O55 + SIgA—piglets challenged with *E. coli* O55 preincubated with SIgA; O55 + dg-SIgA—piglets challenged with *E. coli* O55 preincubated with deglycosylated SIgA. Piglets were fed as long as they were eating and (**a**) the life span and (**b**) volume of suckled milk was monitored. The differences in survival of piglets in individual groups was assessed significant using Mantel–Cox test confirming *P* value < 0.02.

In a separate experiment, we compared the survival of experimental piglets (three piglets per group) after infection with *E. coli* O55 preincubated with SIgA or dg-SIgA, at doses of *E. coli* O55 and SIgA or dg-SIgA analogous to the above-described experiment (Table 1). In contrast, here we monitored the overall survival (Fig. 7a) and suckling volume (Fig. 7b), which was shown in a previous experiment to be one of most valuable predictors of the piglets’ clinical status (Fig. 2). SIgA preincubation with *E. coli* O55 extended the survival of infected piglets in comparison to the untreated *E. coli* O55 infected group. Deglycosylation of SIgA reduced the survival in the SIgA group by about 30%, but still the piglets survive longer than those infected with untreated *E. coli* O55 (Fig. 7a). Comparison of volumes of suckled cow milk indicated good predictive value in time frames of 5–7 h (Fig. 7b). This experiment further confirmed the importance of SIgA *N*-glycans in the protection of piglets from the enteric infection with *E. coli* O55.

## Discussion

Secretions of the intestinal tract contain high levels of antibodies of species-dependent isotype, which display their protective effect by a variety of mechanisms including the agglutination of particulate antigens, inhibition of bacterial adherence to the epithelial receptors, inhibition of uptake of soluble antigens, and extracellular or intracellular neutralization of biologically active antigens.^3–6^ Antibodies interact with antigens through their specificity, polyreactivity, and glycan-dependent binding.^5,8–16,19–22,45,46^ Antibodies, especially of the IgA isotype, may act, due to the presence of glycans, as decoys effectively inhibiting the binding of microorganisms to corresponding epithelial receptors as demonstrated in vitro in a large number of studies.^8–11,14,47^ Here we demonstrated that human IgA-associated glycans exert a mild protective effect in vivo in unique experimental model of newborn, germ-free piglets mono-infected with necrotoxigenic *E. coli* O55.

Germ-free piglets possess a naïve immune system and lack pre-natally acquired immunoglobulins and other factors; thus, they represent an appropriate and relevant physiological model.^48^ Newborn piglets absorb during the first 3 days not only sow colostrum-derived IgG, essential for their survival, but also many heterologous orally administered proteins including human serum albumin, immunoglobulins, or polyvinyl pyridine of various molecular masses. In the case of porcine IgG given orally at doses from 1 to 8 g per piglet, serum levels were 3–22 mg/ml.^49^ Intestinal vacuolated enterocytes present on the tips of villi are primarily involved in the internalization of macromolecules and their transport into the circulation.^50^ These cells display a short lifespan. After 2–3 days they disappear and are replaced by regular enterocytes, which are not involved in the effective trans-epithelial transport from the gut lumen into the circulation.^41,50^ Examination of the intestinal tract of 4 days old piglets demonstrated the presence of IgA in the epithelial cells of villous tips (Fig. S1) indicating the IgA uptake. Gut closure prevents any transfer of antibodies and other proteins in 1–3 days after the first feeding.^49,51^

Due to the earlier reports concerning the intestinal absorption of proteins, we initiated our piglet experiments at the age of 4 days, when the only source of immunoglobulins is spontaneous production of natural antibodies reaching blood concentration of IgG and IgA <50 and <5 ug/ml, respectively, so the interference with experimentally added antibodies should not be taken in account (SIgA was administered at 20 mg/piglet). In fact, we measured the Ig concentration in the blood of all groups of piglets at day 0 and day 6 after delivery, and the mean serum IgG concentration was 30 and 85 μg/ml respectively (data not shown). The concentrations of serum IgA1 and IgA2 isotypes in SIgA- and dg-SIgA-exposed piglets were <0.1 μg/ml for IgA1 and <0.02 μg/ml for IgA2 and in germ-free piglet was not detected (Fig. S2A). The known fact that germ-free piglets have a low level of anti *E. coli* antibodies in their serum^52,53^ was controlled by inclusion of the control group of piglets infected with *E. coli* O55 only that showed similar kinetics of dying as dg-SIgA but significantly accelerated in comparison with SIgA. Thus, we demonstrated that SIgA display its protective activity locally and not through the transfer from the gut lumen into circulation.

Although the inhibition of attachment of microorganism to corresponding cellular receptors by Ig-associated glycans has been well demonstrated in vitro^11,13,15,20,24,54,55^ using a variety of cell lines, studies that document the protective glycan-dependent functions that have been observed in vivo are very limited. To our knowledge there is only one study available in which *N*-linked glycans associated with SC of SIgA were involved in the protection of mice infected with *S. flexneri* by the respiratory route.^29^ Our results indicate that IgA glycosylation is important for amelioration of disease in the germ-free piglet infection model; their enzymatic removal results in a substantial loss of protection for piglets orally infected with pathogenic *E. coli* O55 when compared to the native SIgA. The enzymatic removal of *N-*linked glycans has not substantially diminished the ability of such SIgA preparation in bacterial agglutination. Due to the high degree of heterogeneity of *N*-linked glycans on SC, α H chains, and J chain,^11,20,44^ we have not determined the precise structure of glycans involved. Nevertheless, former structural studies of SIgA-associated glycans revealed a remarkable degree of heterogeneity with respect to the IgA subclass differences, the number of side-chains and their sites of attachment, and variability in the primary structures.^11,20,44^ For the first time we show that Fab as well as Fc-SC have the ability to protect against pathogenic *E. coli* as well as SIgA. Nevertheless, the protection correlates neither with binding nor with agglutination ability suggesting that it is independent of these two phenomena. One possible explanation includes glycan-dependent IgA binding to *E. coli* structures expressed only in the gut environment, or interaction of SIgA with host factors such as mucus or epithelial cells which protect via up-regulation of barrier functions and innate immunity.^56^

These findings are of considerable biological importance because different microorganisms interact with diverse epithelial receptors, which may display structural similarity to SIgA-linked glycans. Importantly, due to differences in their structure, including the composition and possibilities to form various glyosidic bonds among individual component monosaccharides, the combinatorial possibilities are enormous for six commonly present monosaccharides. Experimental analysis identified about 250 different glycoforms on SIgA from saliva samples.^57^ Because SIgA retains its in vitro ability to react with *E. coli* O55 even after the removal of *N-*linked glycans but abolishes its protective activity, it is obvious that the SIgA-associated glycan play an important role. Further structural studies of glycans associated with SIgA are likely to lead to the identification of those involved in the species-specific inhibition of attachment to corresponding epithelial cells.

## Methods

### Reagents

All chemicals, unless otherwise specified, were purchased from Sigma (St. Louis, MO). Tissue-culture media and media-supplement were purchased from Invitrogen (Carlsbad, CA).

### Experimental animals and infections

All animal experiments were approved by the Ethical Committee of the Institute of Microbiology, Czech Academy of Sciences, according to guidelines in the Animal Protection Act.

Pregnant gilts (Minnesota miniature/Vietnam-Asian-Malaysian crossbred pigs^58,59^ were anesthetized at the 112th day of gestation and their fetuses were obtained by hysterectomy under aseptic procedures as described previously.^58,59^ Day of gestation (114th day) was calculated from the day of mating. Neonatal piglets were transferred and maintained in sterile isolator units containing an exterior heat source. The piglets were fed with autoclaved cow milk,^48^ where high temperature denature all proteins including IgG. Each piglet was labeled by ear notching.

Two days after birth, piglets were randomly divided into six groups of five piglets and each group was kept in individual sterile insulator. On the day 4, test animals were infected by suckling of 5 ml of 5% glucose containing *E. coli* O55 pre-incubated with SIgA preparation according to Table 1. All animals used for colonization by *E. coli* O55 were subsequently feed with 10 ml of autoclaved cow milk.

All animals were periodically fed each 3 h and the amount of sucked milk, body temperature, stool appearance/condition and animal behavior were recorded. At the same time, the stool was collected. The first two feedings included supplementary feeding by experimental substances according to animal group and above-described design: (1) only milk, (2) 5 mg of SIgA, (3) 4.5 mg of dg-SIgA, (4) 2.0 mg of Fab, and (5) 3 mg of Fc-SC, respectively.

Piglets were euthanized 35 h after challenge, and lymphoid tissues and blood were collected or frozen for subsequent studies. In survival experiment, piglets were kept alive for monitoring of survival time.

### Inoculation dose of *E. coli O55* incubated with individual SIgA preparations

Samples containing 2.5 × 10^6^ *E. coli* O55 were prepared for each group individually, and were incubated for 1 h with immunoglobulin preparations (125 mg for SIgA and dg-SIgA or 62.5 mg for Fab and Fc-SC or left untreated). Further, samples were used to prepare five doses–each containing 4 × 10^5^ *E. coli* O55 and diluted with 5 ml of sterile glucose and packed in Falcone tube. These preparations were directly used for piglet oral inoculation, and piglets were fed with milk into saturation (Table 1).

### Purification of human milk SIgA^37^

IgA from ammonium sulfate precipitate of pooled human colostrum/milk dissolved in PBS was purified using 25 × 1000 mm Sepharose 6B column equilibrated with buffer (50 mM Tris, 0,9 % NaCl, 10 mM CaCl_2_, 10 mM MgCl_2_, pH 7.4) using NGC 10 system (Bio-Rad Laboratories, Hercules, CA) at flow rate 1 ml/min. Collected fractions were concentrated on 50 kDa cutoff ultracentrifugal filters Amicon Ultra (Millipore, Billerica, MA).

### SIgA cleavage and fragments purification (IgA Fc-SC and Fab)

200 mg of purified SIgA was mixed with 1 mg of recombinant *C. ramosum* protease and incubated at room temperature overnight. The Fc-SC and Fab fragments were separated on 15 × 750 mm Superdex 200 chromatography equilibrated with PBS on NGC 10 system. Fractions with recombinant protein were concentrated by 10 kDa cutoff ultracentrifugal filters Vivaspin (Millipore).

### Preparation of deglycosylated SIgA (dg-SIgA)

150 mg of purified SIgA was treated with 250,000 U of EndoH (NEB, Ipswich, MA) for 72 h at 37 °C, according to manufacturer instructions, the sample was desalted by 50 kDa cutoff ultracentrifugal filters (Millipore), and the sample was treated with 375,000 U of PNGaseF (NEB) according to manufacturer instructions under non-denaturing conditions for 36 h at 37 °C. The dg-SIgA was characterized on SDS-PAGE followed by Coomassie blue staining. The rate of *N*-glycans removal was determined on western blot with biotin-conjugated *Galanthus nivalis* lectin (Vector Laboratories, Burlingame, CA) and HRP-neutravidin as the chemiluminescence reduction recorded on cooled CCD camera.

### Electrophoretic analysis of IgA preparations

SDS-PAGE separated proteins were stained with Coomassie brilliant blue G-250 solution. The electrophoresis was performed under reducing (using sample buffer with 2-mercaptoethanol) or non-reducing conditions. The concentration of all IgA preparations used in animal experiments was determined by Bradford Protein Assays (Bio-Rad Laboratories).

### Quantification of IgA1, IgA2 in the stool samples

The concentration of IgA1 and IgA2 in stool was determined by ELISA.^60^ ELISA plates were coated overnight with 1 µg/ml F(ab’)_2_ fragment of goat IgG anti-human IgA (Jackson Immunoresearch Labs, West Grove, PA) in PBS. Coated plates were blocked with 1% BSA in PBS with 0.05% Tween-20. Serial 2-fold dilutions of duplicate samples and standards in blocking solution were incubated overnight. The bound IgA1 and IgA2 were detected using mouse monoclonal anti-IgA1 and anti-IgA2 antibodies and followed by goat anti-mouse IgG HRP-conjugated antibody. As a HRP substrate was used OPD. The amount of IgAs in the tested samples was calculated by interpolating the OD 490 values on calibration curves. As a standard, myeloma IgA1 for IgA1 and myeloma IgA2 for IgA2 was used.

### Intestinal histology

Intestinal tissue samples were dissected, formalin-fixed, and paraffin-embedded. Sections were stained with hematoxylin and eosin (H&E) and classified by a single pathologist without prior knowledge of clinical parameters using BX43 microscope equipped with CCD camera.

### Enumeration of bacterial load in individual tissues and stool

Stool samples were collected every 3 h when piglets were fed. The stool was weighted, diluted in sterile PBS, and inoculated on LB agar plates. Samples of individual tissues were collected after termination of experiment, homogenized, and after serial dilution in sterile PBS inoculated on LB agar plates. After 16 h cultivation at 37 °C colonies were counted and after correction to dilution factor expressed as CFU/mg. CFU for individual piglets are shown. Remaining samples of stool and individual tissues for IgA1 and IgA2 assessment were immediately frozen in liquid nitrogen and kept frozen at −80 °C.

### Analysis of inflammatory markers in gut tissue samples by quantitative PCR

Tissue biopsies were collected immediately after the animals euthanize procedure and stored in RNAlater (Thermo Fisher Scientific, San Jose, CA) until further use. Total RNA was isolated from tissue biopsies using TRI Reagent (Sigma). Tissue samples (approx. sample weight in mg) were homogenized in 600 μl of TRI Reagent in Lysing Matrix D tubes (MP Biomedicals, Irvine, CA) using FastPrep homogenizer (MP Biomedicals). Total RNA was isolated according to the manufacturer’s protocol. The concentration of RNA was measured by Nanodrop 1000 (Thermo Fisher Scientific).

RNA samples were treated with TURBO DNA-free Kit (Thermo Fisher Scientific), and 500 ng of total RNA was reverse transcribed using oligo(dT)_20_ primers and SuperScript IV Reverse Transcriptase (Thermo Fisher Scientific). The resulting cDNA served as a template in quantitative PCR using a CFX96 Real-Time PCR detection system (Bio-Rad). Quantification of the gene amplicons was detected using SYBR green RT PCR (SG PCR Master Mix Generi Biotech, Hradec Kralove, Czech Republic). Each PCR reaction was performed in triplicates. The amplification protocol was as follows: 3 min at 95 °C followed by 39 cycles at 94 °C for 30 s, 59 °C for 35 s, and 72 °C for 50 s. Melting curves analysis was performed for confirmation of specificity and uniformity of PCR products. Primers were designed using Primer3Plus (Andreas Untergasser, Germany). Changes in gene expression were calculated using the 2^−ΔΔCT^ method.^61^ Quantitative measurements were normalized using peptidylprolyl isomerase A (PPIA) mRNA as the reference gene marker. Changes in mRNA levels in different groups are shown as the fold change of expression related to control (germ-free piglets). Data were expressed as mean ± SEM of the values obtained in all experiments.

### Agglutination assay

The immunoglobulin preparations at 2 mg/ml were titrated by twofold dilutions in 0.01 M phosphate-buffered saline (pH 7.2; PBS) supplemented with Ca^2+^/Mg^2+^ in 96-well U-shaped microtiter plates (Thermo Fisher Scientific). *E. coli* O55 was added to final concentration at 2 × 10^9^ CFU/ml in PBS. The plates were shaken and incubated at 37 °C for 30 min and then at 4 °C overnight. Agglutination was read by eye. The reciprocal of the highest dilution giving visible agglutination was recorded as the agglutination titer.

### Flow cytometry analysis of IgA binding to *E. coli* O55

One hundred μg of SIgA and derived fractions were conjugated with DyLight 488 and characterized according to manufacturer’s instructions (Thermo Fisher Scientific). About 5 μg of individual DyLight 488-labeled SIgA preparations, previously confirmed as oversaturating the bacteria, were incubated with 10^6^ CFU of *E. coli* O55 for 30 min, followed by washing with 1% FBS in PBS and analyzing by flow cytometry using SP6800 Spectral Cell Analyzer (Sony Biotechnology, San Jose, CA).

### Immunohistochemistry

Intestinal tissue samples were embedded in Tissue-Tek O.C.T., snap-frozen in liquid nitrogen via isopenthane, and stored at −70 °C. The 6 µm cryosections were air-dried, fixed for 5 min in acetone, and stored at −20 °C. The sections were incubated with Pap Pen and FITC conjugated anti-human IgA heavy chain-specific mAb (clone M 24 A CBL-114F) was added at a dilution 1:30, incubated overnight at 4 °C, and washed three times with PBS. Control sections, were treated with PBS without mAb. All sections were mounted in medium with DAPI (Invitrogen). Specimens were observed in an Olympus BX 40 microscope.

### Statistical analysis

Statistical analyses were performed using GraphPad Prism version 8 (GraphPad Software, San Diego, CA). Differences between groups were evaluated by One-way ANOVA with Dunnett´s Multiple Comparison test. A *p* values < 0.05 was considered significant. Data are presented as mean ± SD.

## Supplementary information

Supplementary Information
