# Supplementary material for: Secretory IgA N-glycans contribute to the protection against E. coli O55 infection of germ-free piglets
Source: Mucosal Immunol. 2020 Sep 24;14(2):511–22. doi: 10.1038/s41385-020-00345-8 (PMC7946640; doi:10.1038/s41385-020-00345-8)
Supplement: Supplementary file 1 — Supplementary Information [file 41385_2020_345_MOESM1_ESM.pdf]

Secretory IgA *N*-glycans contribute to the protection against *E. coli* O55 infection of germ-free piglets.

Leona Raskova Kafkova, Diana Brokesova, Michal Krupka, Zuzana Stehlikova, Jiri Dvorak, Stepan Coufal, Alena Fajstova, Dagmar Srutkova, Katerina Stepanova, Petra Hermanova, Renata Stepankova, Ivo Uberall, Jozef Skarda, Zdenek Novak, Luca Vannucci, Helena Tlaskalova Hogenova, Zuzana Jiraskova Zakostelska, Marek Sinkora, Jiri Mestecky, Milan Raska

Supplementary Table 1. Scoring of clinical status of individual piglets.

Piglets	Temperature	Volume per feeding	Stool	Viability
**Normal**	1	1	1	1
	1	2	1	1
**O55**	4	2	2	3
	4	2	2	2
	4	3	2	5
	3	2	2	2
	3	2	2	4
**O55 + Fc-SC**	2	1	2	1
	3	3	2	4
	2	1	2	1
	2	3	2	3
	2	1	2	3
**O55 + SIgA**	3	1	1	2
	3	1	1	2
	3	3	1	3
	3	1	1	1
	3	1	1	2
**O55 + Fab**	2	2	1	1
	2	2	1	1
	3	2	1	3
	2	2	1	1
	2	2	1	2
**O55 + dg-SIgA**	4	2	1	4
	4	1	1	4
	4	2	2	4
	5	3	2	5
	4	3	2	5

Individual parameters scaling is detailed in Table 2. Parameters were used for calculation of weighted mean for each group as summarized in **Figure 2**.

Supplementary Table 2. Genotyping of *E. coli* O55 used in experiments.

F antigen - E.coli					
**Fimtype**	**%Identity**	**Query/HSP length**	**Contig**	**Position in contig**	**Acc. No.**
fimH39	100.00	489 / 489	NODE_11_length_166574_cov_29.242607	34278..34766	KJ096308.1
F4	No hit found				
F5	No hit found				
F6	No hit found				
F18	No hit found				
F41	No hit found				
aeaA	No hit found				

O (LPS) type genes						
**Gene**	**Serotype**	**Identity**	**Template / HSP length**	**Contig**	**Position in contig**	**Acc. No.**
wzy	O55	97.65	978 / 978	NODE_263_length_156576_cov_28.013201	32640..33617	AB353132
wzy	O55	97.65	978 / 978	NODE_263_length_156576_cov_28.013201	32640..33617	CP003109
wzx	O55	98.59	1278 / 1278	NODE_263_length_156576_cov_28.013201	31345..32622	JH958641

H type genes						
**Gene**	**Serotype**	**Identity**	**Template / HSP length**	**Contig**	**Position in contig**	**Acc. No.**
fliC	H4	100	1050 / 1050	NODE_7_length_110642_cov_28.547831	31034..32083	AJ605764

Toxin genes						
**Virulence factor**	**Identity**	**Query / Template length**	**Contig**	**Position in contig**	**Protein function**	**Acc. No.**
STa	No hit found					
STb	No hit found					
LT	No hit found					
stx1	No hit found					
stx2	No hit found					
hlyA	No hit found					

Virulence genes for Escherichia coli						
**Virulence factor**	**Identity**	**Query / Template length**	**Contig**	**Position in contig**	**Protein function**	**Acc. No.**
CNF	100				Cytotoxic necrotizing factor 1	
cdtB	100	810 / 810	NODE_65_length_4237_cov_21.189993	1338..2147	Cytolethal distending toxin B	AY365044
f17A	100	546 / 546	NODE_48_length_9350_cov_38.190052	1098..1643		L43373
f17G	100	1032 / 1032	NODE_48_length_9350_cov_38.190052	4988..6019		CP001162
iroN	99.95	2178 / 2178	NODE_370_length_39709_cov_34.301922	10819..12996	Enterobactin siderophore receptor protein	AE014075
iss	98.98	294 / 294	NODE_58_length_2362_cov_71.282387	1758..2051	Increased serum survival	CP001509
lpfA	100	573 / 573	NODE_2_length_174238_cov_29.314823	143758..144330	Long polar fimbriae	AY646923
mchB	100	294 / 294	NODE_370_length_39709_cov_34.301922	34109..34402	Microcin H47 part of colicin H	AE014075
mchC	100	1551 / 1551	NODE_370_length_39709_cov_34.301922	32287..33837	MchC protein	AE014075
mchF	100	2115 / 2115	NODE_370_length_39709_cov_34.301922	28293..30407	ABC transporter protein MchF	AE014075
mcmA	100	279 / 279	NODE_370_length_39709_cov_34.301922	27761..28039	Microcin M part of colicin H	AJ586887

Enterobacteriaceae plasmids						
**Plasmid**	**Identity**	**Query / Template length**	**Contig**	**Position in contig**	**Note**	**Acc. No.**
IncFIB(AP001918)	99.56	682 / 682	NODE_124_length_4137_cov_27.241480	1606..2287		AP001918
IncFII(pSE11)	95.45	264 / 264	NODE_56_length_14019_cov_18.551752	10970..11226	pSE11	AP009242

Gram Positive plasmids						
Plasmid	Identity	Query / Template length	Contig	Position in contig	Note	Acc. No.
No hit found						

MLS - Macrolide, Lincosamide and Streptogramin B					
Resistance gene	Identity	Query/HSP	Contig	Position in contig	Acc. No.
mdf(A)	98.78	1233/1233	NODE_5_length_336678_cov_27.747412	30218..31450	Y08743

Green color indicates matching of O55 sequence with database target (identity), light green indicates partial matching, and magenta indicates no matching. The search was performed by the use of ResFinder-3.1 Server, FimTyper-1.0 Server.[1] PlasmidFinder-2.0 Server, and SerotypeFinder-2.0 Server all available on https://cge.cbs.dtu.dk, and by the use of https://bitbucket.org/genomicepidemiology/fimtyper.

Supplementary Figure 1. Detection of IgA within duodenal vacuolated enterocytes.

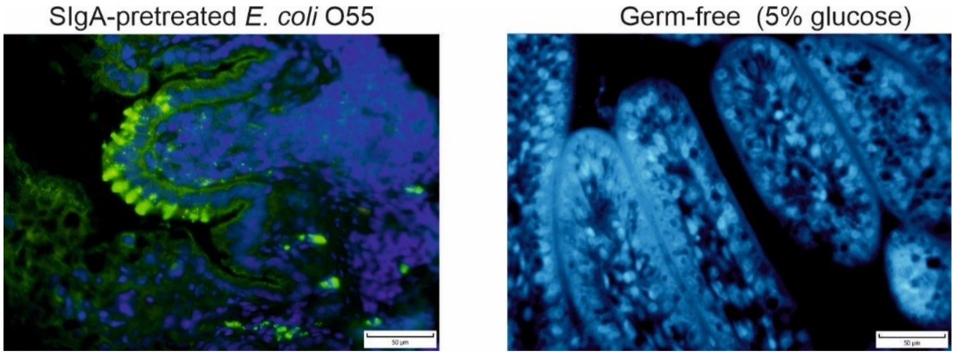

Frozen tissue section of duodenum from piglet infected with *E. coli* O55 preincubated with SIgA and from germ-free piglet. Tissue sections were stained with human IgA-specific FITC-labeled mAb. Nuclei were stained with DAPI. Left section from piglet infected with *E. coli* O55 incubated with SIgA, right section from germ-free piglet not exposed to human SIgA.

Supplementary Figure 2. Human IgA in the blood and intestine in piglets infected with *E. coli* O55-incubated with SIgA and dg-SIgA.

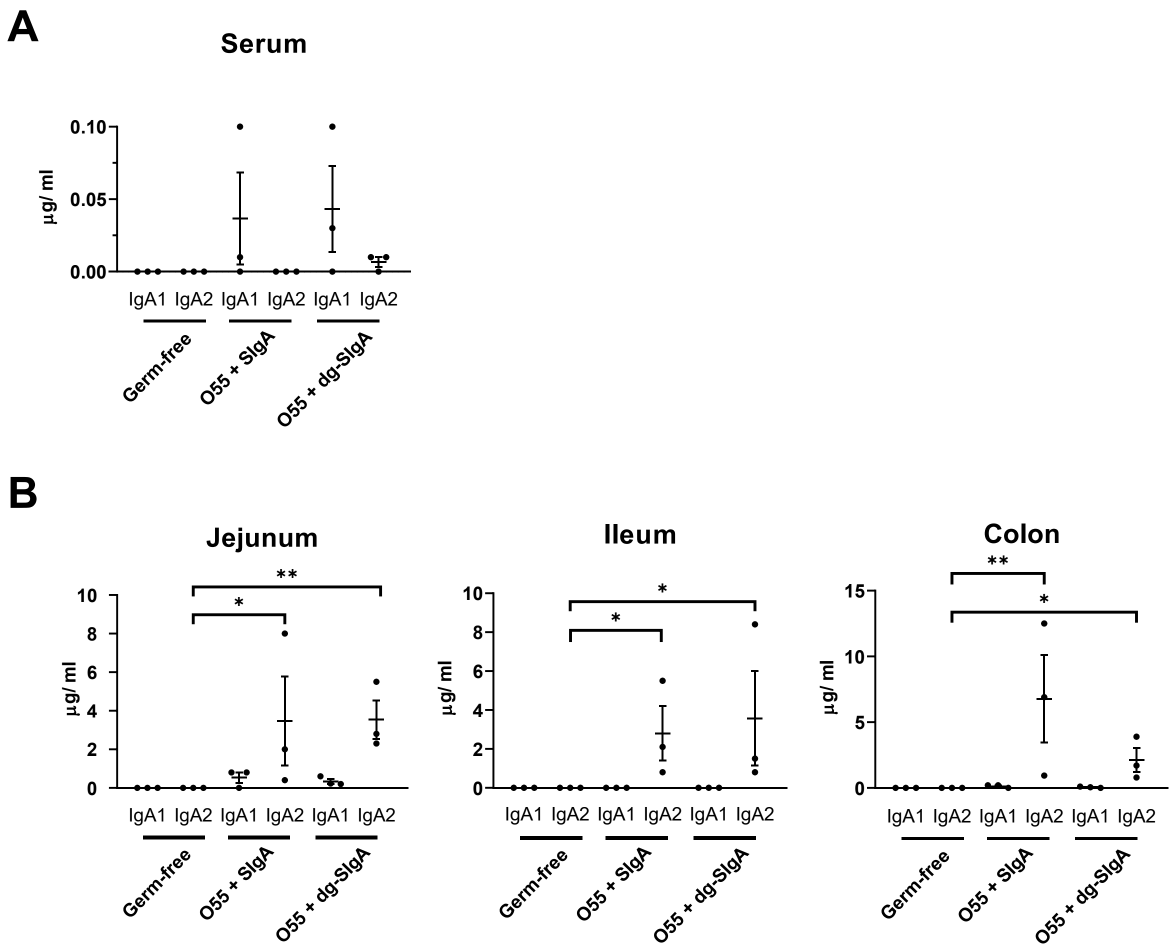

A) The concentration of IgA1 and IgA2 in the serum of three randomly selected piglets per group. The level of IgA1 was increased in piglets infected with *E. coli* O55 preincubated with both SIgA and dg-SIgA. The level of IgA2 was slightly increased in group infected with *E. coli* O55 preincubated with dg-SIgA. **B)** The concentration of IgA1 and IgA2 in the lumen of jejunum, ileum, and the colon. IgA2 was increased in jejunum, ileum, and colon in the group of piglets infected with *E. coli* O55 preincubated with SIgA and dg-SIgA. IgA1 was slightly increased (ns) only in jejunum of piglets infected with *E. coli* O55 preincubated with SIgA and dg-SIgA. There is no significant difference between the amounts of IgA2 in jejunum of piglets infected with *E. coli* O55 preincubated with SIgA and dg-SIgA. Data are means ± SD. P values were calculated using Kruskal-Wallis test with Dunn-Bonferroni post-hoc test. * $p < 0.05$, ** $p < 0.01$.

Supplementary Figure 3. Determination IgA and bacteria content in the stool during the course of experiment.

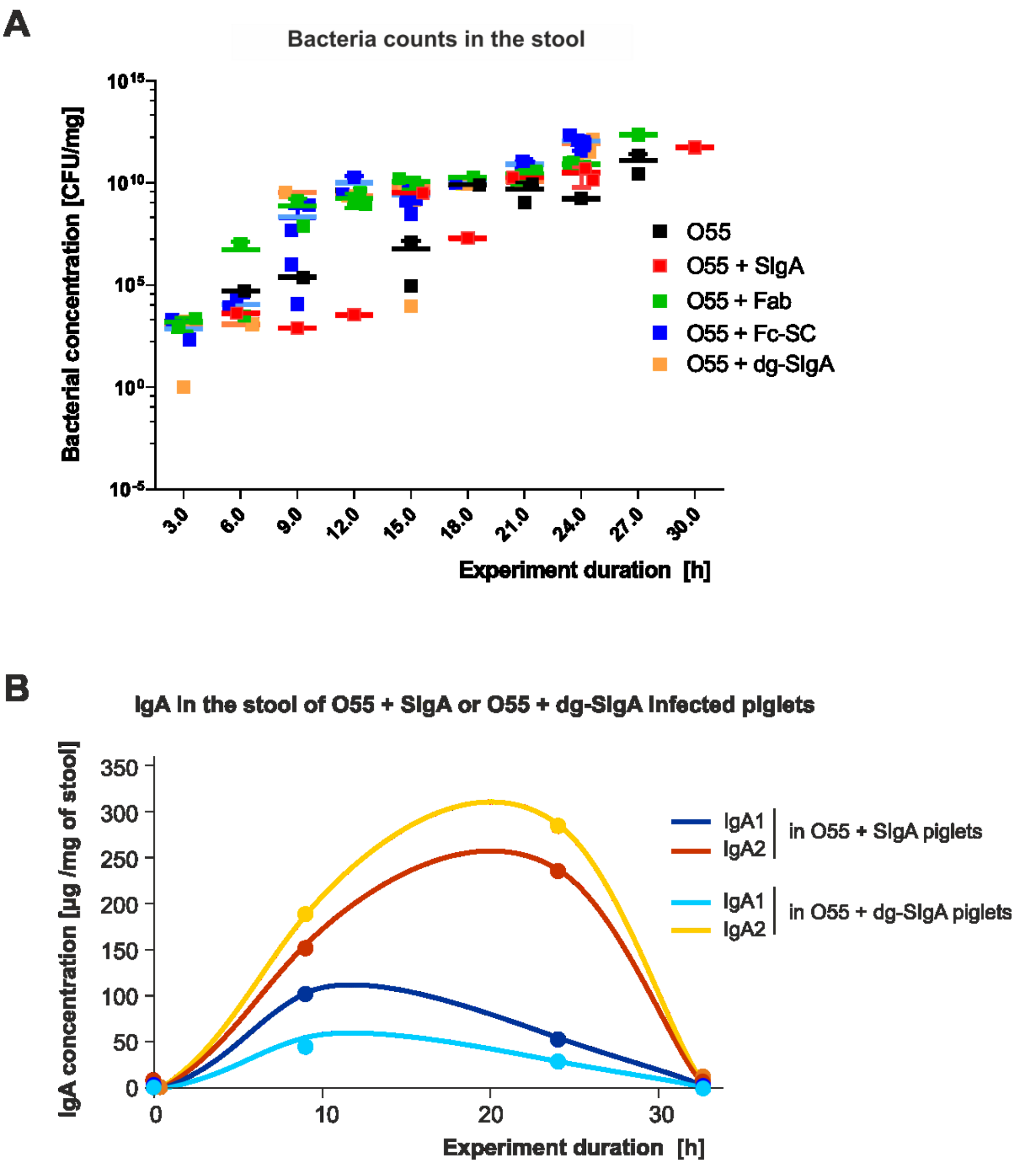

A) Bacterial load was determined from stool samples taken at indicated time points. The numbers of stool samples analyzed varies (n = 1 - 4) and for individual time point it corresponds to numbers of group-specific colored squares. The bacterial load is

expressed as means bacterial colony forming unite count calculated on agar plates (CFU/mg of intestine content). **B**) IgA1 and IgA2 were determined in stool samples of piglets infected with *E. coli* O55 + SIgA or with *E. coli* O55 + dg-SIgA taken before, 9, 24 hours after infection, and at the termination of the experiment (35 h after infection). Analyzed were samples from 1 to 4 piglets per group. IgA levels were determined by ELISA assay using monoclonal antibodies against IgA1 and IgA2 and expressed as means. Because the number of stool samples differs among groups and time points, we did not use statistical analysis here. At the termination of experiment the concentration of individual IgAs was below 13 μg/mg of stool. The curves represent polynomic approximation of time course of individual IgA sequestration.

Supplementary Figure 4. ***E. coli*** **O55 morphology and agglutination assay detail.**

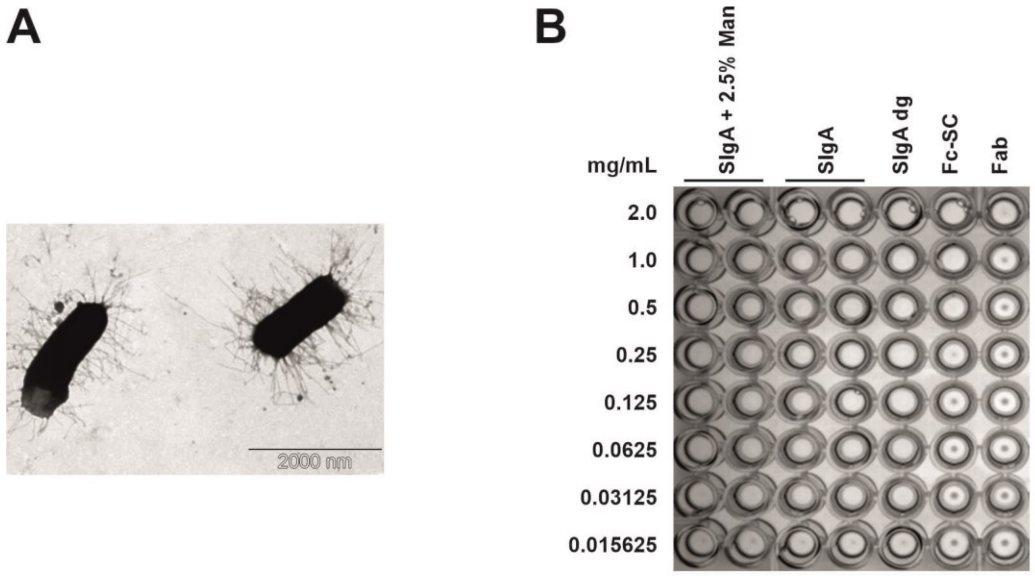

A) *E. coli* O55 was grown in LB agar at 37°C for 16 h and thereafter culture was placed in 4°C overnight. Bacteria cells were harvested by mild centrifugation, washed, and fixed and mounted for observed by transmission electron microscopy. **B**) Detail of macroscopic agglutination assay performed with SIgA preparations at 2 mg/ml incubated with 2x10^9/ml *E. coli* O55 at 37°C followed by 4°C incubation overnight. Sedimented non-agglutinated bacteria pellets are visible.

Supplementary Figure 5. Binding of SIgA preparations to *E. coli* O55.

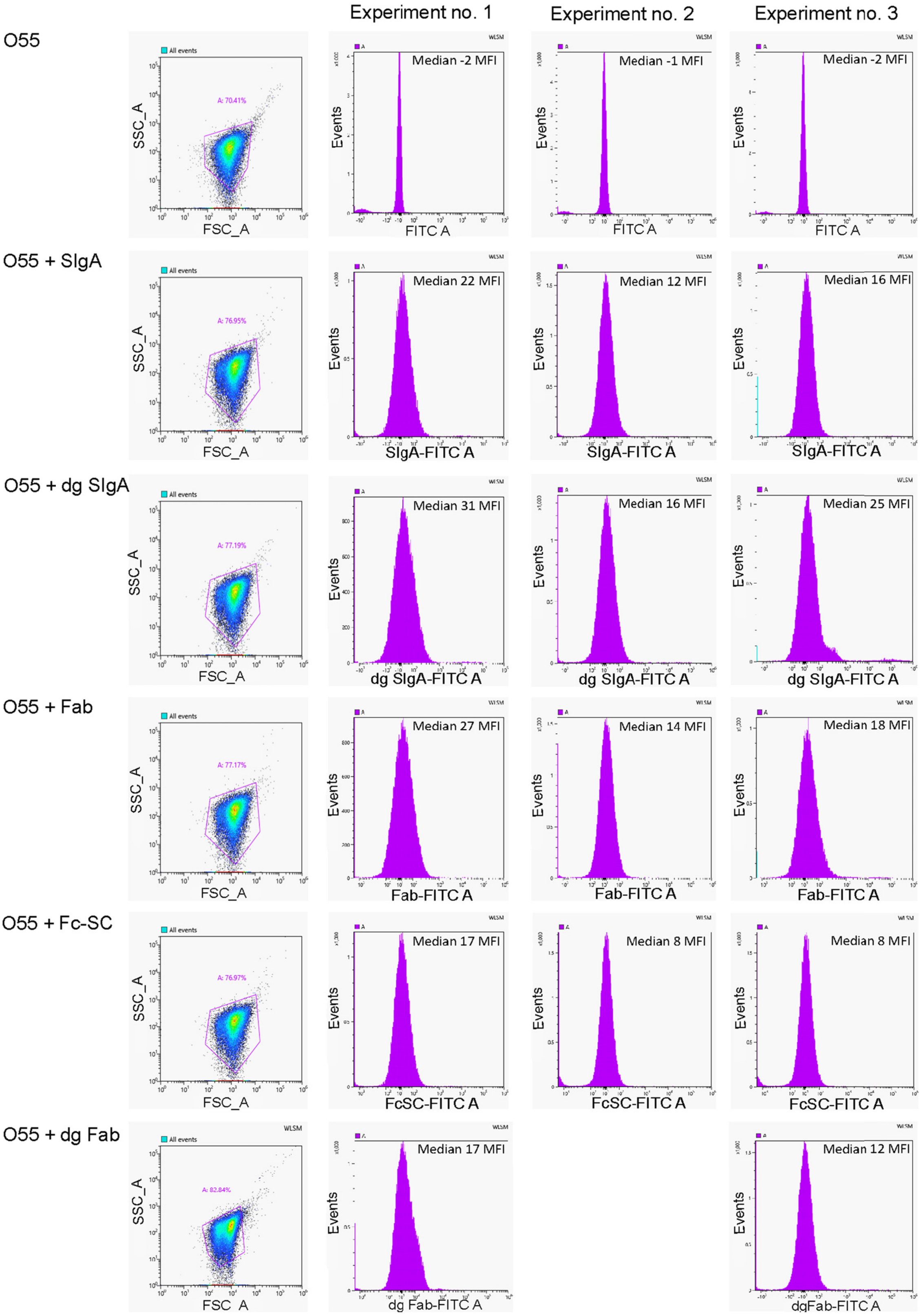

E. coli O55 was incubated with individual SIgA preparations conjugated with DyLight 488, washed, and analyzed by flow cytometry. Individual plots are provided with median MFI.
